# Dimensional stability of 3D-printed edentulous and fully dentate hollowed maxillary models over periods of time

**DOI:** 10.1186/s12903-024-04256-z

**Published:** 2024-04-26

**Authors:** Mohammad Zarbah, Omir Aldowah, Nasser M. Alqahtani, Saud Ali Alqahtani, Maha Alamri, Reem Alshahrani, Noaf Mohsinah

**Affiliations:** 1 Department of Prosthetic Dental Science, King Khalid University, Abha, Saudi Arabia; 2 Department of Prosthetic Dental Science, Najran University, Najran, Saudi Arabia; 3 Dental Intern, King Khalid University, Abha, Saudi Arabia

**Keywords:** 3-D printed models, Dimension stability, Aging effects

## Abstract

**Background:**

Dental casts made utilising digital workflow are becoming more common because to their speed and cost savings. However, studies on their dimensional accuracy over time with diverse designs are missing.

**Objective:**

The aim of this in vitro study was to assess the dimensional stability of 3D-printed edentulous and fully dentate hollowed maxillary models with 50-micrometer resolution over 1 day, 14 days, and 28 days using surface matching software.

**Methods:**

Scanned edentulous and fully dentate maxillary typodont models were used as references. The models were scanned by a desktop lab scanner of 15-micrometer accuracy (D900, 3Shape). Then, the files were used in designing software (Meshmixer, Autodesk) to create hollowed maxillary casts. Fifteen edentulous and 15 fully dentate (total of 30) models were printed using a DLP lab printer (Cara print 4.0, Kulzer). The 3D-printed models were scanned using the same desktop lab scanner of 15-micrometer accuracy at intervals of baseline days, 1 day, 14 days, and 28 days to assess the effect of aging (*n* = 120). The dimensional changes were quantified and compared using the root mean square (RMS) method, expressed in micrometres (µm). The study employed repeated measures analysis of variance (ANOVA) to assess and compare the root mean square (RMS) values across the variables. The data was analysed using SPSS (26, Chicago, Illinois, USA).

**Results:**

The RMS of the edentulous models rapidly increased from a mean value of 0.257 at the beginning of the study to 0.384 after twenty-eight days. However, the mean RMS values for the dentate models did not change much over the four intervals. It varied only from 0.355 to 0.347. The mean values for edentulous patients increased from 0.014 to 0.029 during the period from baseline to twenty-eight days. However, the mean average values decreased for the dentate models from 0.033 to 0.014 during this period. By utilizing ANOVA, mean RMS values increased insignificantly till one day but significantly to fourteen and twenty-eight days. Dentate model mean values differed insignificantly across four intervals. Repeated measures ANOVA for combined and separated data showed no significant differences across edentulous, dentate, and total models over times.

**Conclusion:**

The study revealed changes in the dimensions of 3D-printed edentulous models over a span of 3 and 4 weeks. Caution should be applied when using 3D-printed dental master models for constructing definitive prostheses on edentulous models over a period of 3 to 4 weeks.

## Introduction

Integration of the digital workflow into the field of dentistry for the fabrication of prostheses has made significant progress in recent years. Digital workflows are increasingly used to construct dental models because they reduce the amount of time and money needed for such work, especially if the dental laboratory is located too far from the dental clinic [1, 2]. Even though the rapid development of technology has changed many aspects of digital dentistry, physical dental models, either poured or 3D-printed, are still needed in many laboratory procedures. Currently, not all prostheses are amenable to model-free workflows such as manually veneered fixed dental prostheses (FDPs) as well as finalization of removable partial denture (RPD) frameworks [3]. The 3D printing process is an exciting technique for fabricating dental models from polymers using lab-side or intraorally scanned data [1, 4]. Among the available design for printing the models, hollowed model design is widely used in many cases, as the removal of unnecessary parts would reduce the time and amount of consumed resin material [5]. Compared to traditional stone casts, 3D-printed dental models were believed to be clinically acceptable in regard of accuracy and reproducibility [6]. Additionally, when compared to CAD/CAM milled or 3D-printed models revealed even greater accuracy [7].

The dimensional stability of 3D-printed dental models is clinically important in instances when both conventional and digital workflows are needed. In cases of inadequate access to 3D printers, where delays in service are unavoidable due to a heavier workload, insufficient in-house lab facilities, and shipment requirements of countryside and outreach locations, the dimensional stability of 3D printed models is of crucial significance. Therefore, it is crucial that a 3D-printed model maintains its dimensions while being stored so that the prosthesis can be properly fabricated for accurate seating, acceptance with the patient’s stomatognathic system and the intended treatment, specifically in multiunit prostheses that need a passive fit on insertion, and surgical guides for implants placement [8].

Joda et al. [9] examined the influence of aging on the dimensional stability of 3D-printed dental models. It was carried out on the dentate models with tooth-supported, three-unit FDPs. This study observed a statistically significant alteration in dimensions over time. After 3 weeks of aging under control conditions, a notable decrease in the dimensional stability of the models was noted. 3D-printed dental master models may not be the option to fabricate definitive prostheses 3 to 4 weeks after printing. Similarly, another study found that aging had an impact on dentate 3D-printed models with different storage methods [10]. Both previous studies assessed the dimensional accuracy of the fully filled and hexagon-filled dentate models over a period of time. Considering the widespread use of hollowed model designs and the findings of Shin et al. [5] indicating that such models exhibit lower accuracy, it could be necessary that one emphasizes the examination of dimensional stability of hollowed 3-D printed models. To the best of the authors’ knowledge, studies comparing the effect of aging on edentulous and fully dentate hollowed maxillary 3D-printed models are lacking.

Hence, the aim of this in vitro study is to assess the dimensional stability of 3D-printed edentulous and fully dentate hollowed maxillary models with 50-micrometer resolution over 1 day, 14 days, and 28 days using surface matching software.

## Methodology

**Fig. 1:**
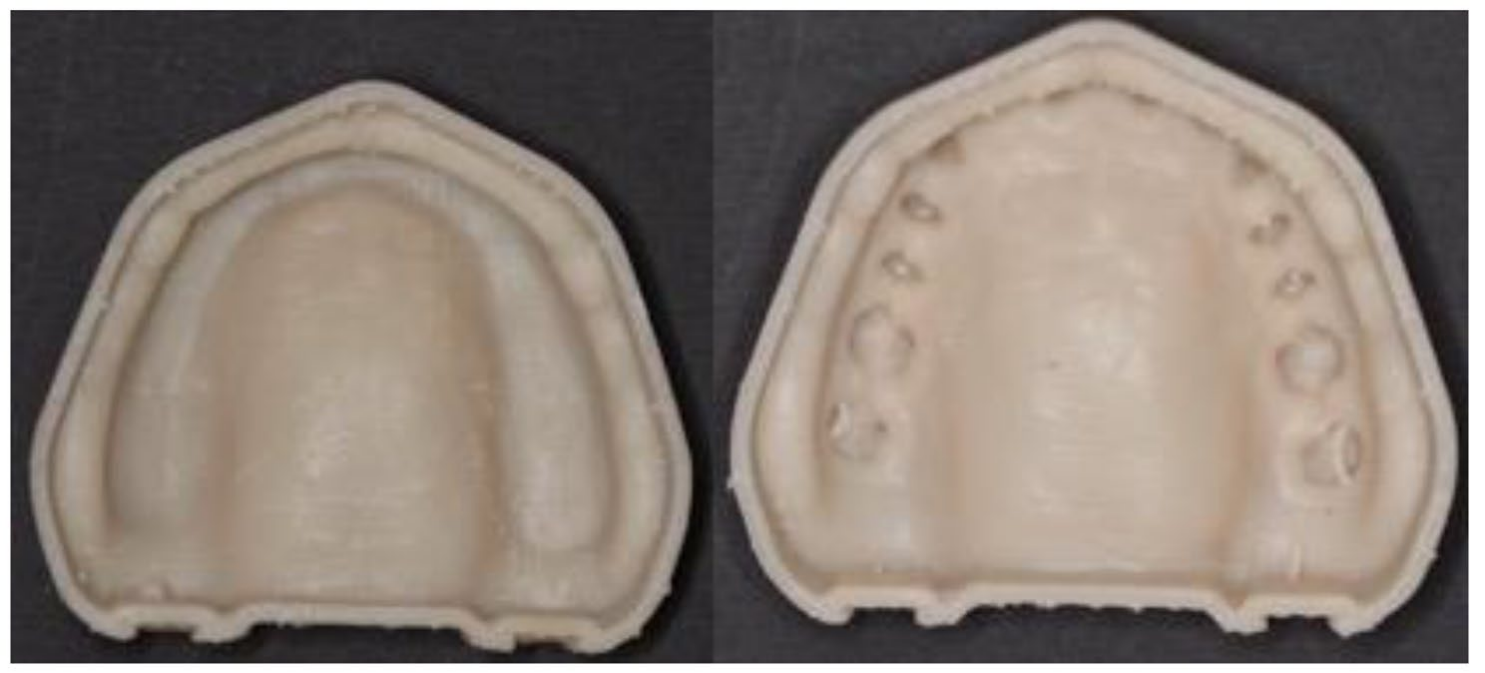
Hollowed models

This study was approved by the research ethics committee at King Khalid University, Abha, Reference No. 443/40-50397-DS. Scanned edentulous and fully dentate maxillary typodont models without defects (Frasaco, GmbH) were used as control negative scan. A desktop lab scanner of 15-micrometer accuracy (D900, 3Shape) was first calibrated and then the control negative models were scanned by a single operator according to the scanning method and sequence recommended by the manufacturer. The exported files were saved in standard triangular language (STL) and were utilized as baseline models. Then, the files were used in designing software (Meshmixer, Autodesk) to create hollowed maxillary casts. Fifteen edentulous and 15 fully dentate (total of 30) models were printed using a DLP lab printer (Cara print 4.0, Kulzer) with Model V2 Resin material (Formlabs, Somerville, MA, USA), Fig. 1. The printed edentulous and fully dentate maxillary hollowed models were considered as control positive models and scanned by the same operator with the same protocol applied for the the control negative models. The models were stored in dry storage at room temperature. The 3D-printed models were scanned using the same desktop lab scanner of 15-micrometer accuracy at intervals of baseline day, 1 day, 14 days, and 28 days to assess the effect of aging (Total of 120 scans). The scanned files were saved in the format of STL files. The files were used in surface matching software (Geomagic design x, 3D Systems, Rock Hill, SC, USA) according to the best-fit alignment method, and then the dimensional stability of the printed models was assessed. The dimensional accuracy was measured and compared by the root mean square (RMS, in µm). Repeated measures^1^ design with interval factors (baseline, one day, fourteen days, and twenty-eight days) as among the groups and edentulous and dentate models as between the groups were employed. The ‘Simple’ method with ‘baseline’ as a reference was used for comparison. The data was analyzed using SPSS (26, Chicago, Illinois, USA)

## Results

The descriptive statistics (mean and standard deviation) of the RMS values for the hollowed dentate and edentulous models are presented in Table 1. The RMS of the edentulous models rapidly grew from a mean value of 0.257 at the beginning of the study to 0.384 after twenty-eight days. However, the mean RMS values for the dentate models did not change much over the four intervals. It varied only from 0.355 to 0.347. Consequently, the average RMS values changed from 0.306 at baseline to 0.365 at twenty-eight days. The descriptive statistics of the averages are shown in Table 1. The mean values for edentulous patients increased from 0.014 to 0.029 during the period from baseline to twenty-eight days. However, the mean average values decreased for the dentate models from 0.033 to 0.014 during this period. These two series of means moved in opposite directions. Therefore, the means of the total sample did not change much and changed only from 0.023 to 0.021 during the baseline to twenty-eight days.

**Table 1:** Comparison of mean RMS values and average values among four intervals and two groups

Factor	Group	No.	Baseline	One day (24 h)	Fourteen days	Twenty-eight days	Average
RMS	Edentulous	15	0.257 ± 0.11^a^	0.300 ± 0.09^a^	0.368 ± 0.11^b^	0.384 ± 0.06^b^	
	Dentate	15	0.355 ± 0.05^a^	0.355 ± 0.05^a^	0.353 ± 0.07^a^	0.347 ± 0.08^a^	
	Total	30	0.306 ± 0.10^a^	0.327 ± 0.08^a^	0.360 ± 0.09^b^	0.365 ± 0.07^b^	
Average	Edentulous	15	0.014 ± 0.02^a^	0.013 ± 0.03^a^	0.030 ± 0.03^a^	0.029 ± 0.05^a^	0.021
	Dentate	15	0.033 ± 0.03^a^	0.033 ± 0.03^a^	0.018 ± 0.02^a^	0.014 ± 0.02^a^	0.024
	Total	30	0.023 ± 0.03^a^	0.023 ± 0.03^a^	0.024 ± 0.03^a^	0.021 ± 0.04^a^	

The superscript shows statistical significance

**Fig. 2:**
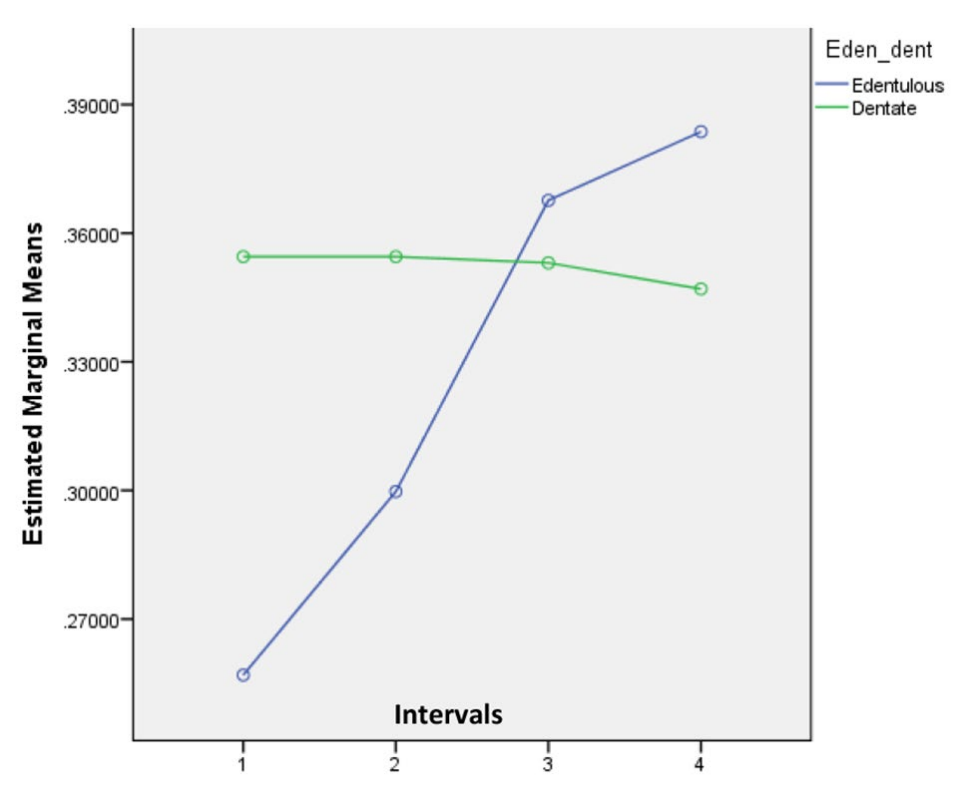
Line graph for mean RMS values of edentulous and dentate models

**Fig. 3:**
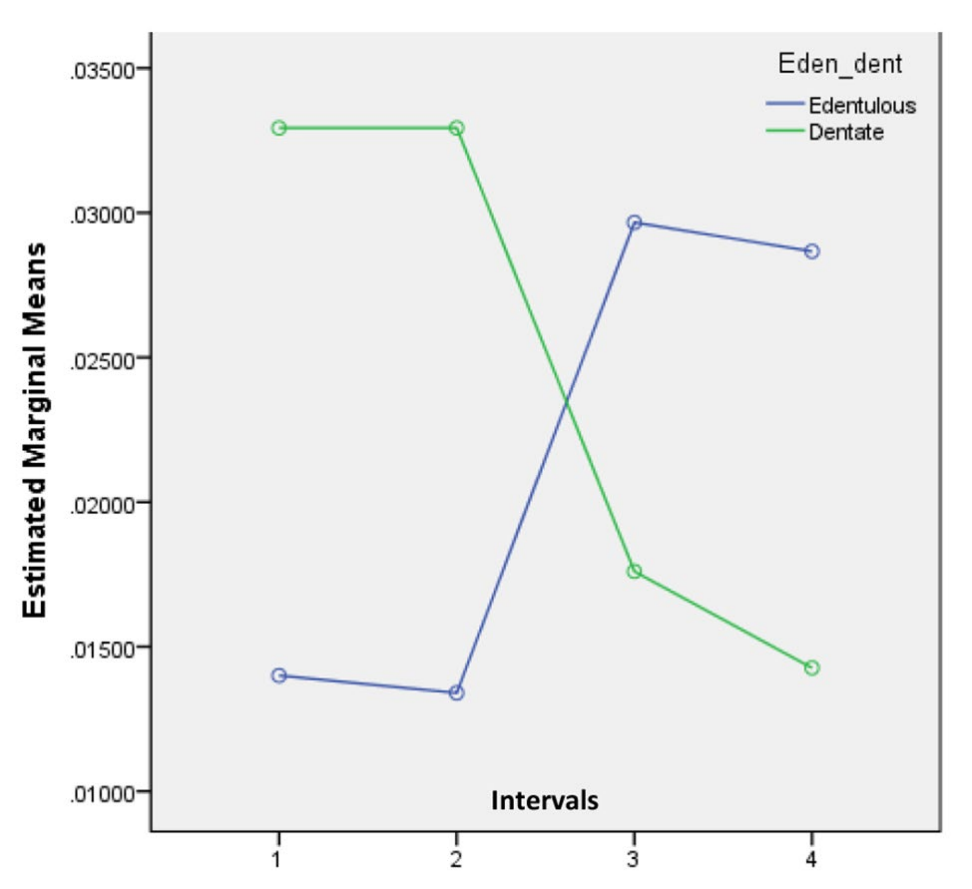
Line graph for mean values of average

The *p* values of intervals and interactions with groups (edentulous and dentate) are presented in Table 2. There was no statistically significant difference between baseline and day one (*p* = 0.241). Nevertheless, the fourteen-day and twenty-eight-day observations were significantly different from the baseline readings (*p* value 0.002 in each). These results are also indicated in the mean values of the total in Table 1. However, the interaction effects between groups and intervals were also significantly different (*p* = 0.002 and 0.001). It is also shown in the graph that the mean RMS values of the edentulous and dentate models were moving in different directions (Fig. 2). The results also showed that there were no effects of the groups (*p* = 0.239) because two mean values of the edentulous models were below and two mean values were above the curve of the mean values of the dentate models. Table 2 Also shows the significance levels (*p* value) of the effect of intervals and the interaction effect of intervals and groups. There was no effect on any of the intervals with reference to baseline data. This should be due to the opposite movements of mean values for edentulous and dentate casts. This trend is also verified by the significant interaction effect of baseline to the fourteen-day interval (*p* = 0.037) and the marginally significant effect of baseline to the twenty-eight-day interval (*p* = 0.058). Furthermore, there was no group effect for the same reason (*p* = 0.647). It can also be seen in Fig. 3. Since there was a significant interaction effect, repeated-measures ANOVA was applied separately for edentulous and dentate casts.

**Table 2:** Effect of intervals and treatment groups (edentulous and dentate) for average observations

Factor	Group		Baseline vs. One-day	Baseline vs. fourteen days	Baseline vs. twenty-eight days
RMS	Edentulous	Intervals (*p* value)	0.241	0.002	0.002
	Dentate	Intervals vs. groups	0.241	0.002	0.001
		*p* value for group effect = 0.239			
Average	Edentulous	Intervals (*p* value)	0.954	0.981	0.814
	Dentate	Intervals vs. groups	0.954	0.037	0.058
		*p* value for group effect = 0.647			

Since there was an interaction effect between group and timeline, the repeated-measures ANOVA was recalculated for each component of the group (edentulous and dentate). The results showed that only edentulous cast observations were significantly affected from baseline to fourteen and twenty-eight days of observation (*p* value = 0.002 each); dentate models changed insignificantly during the intervals (*p* values = 0.921 and 0.536) (Table 3). These significant values are also indicated in the edentulous and dentate rows in Table 1. Moreover, The *p* values for the comparison of the baseline to one day, fourteen days, and twenty-eight days are presented in Table 3. None of the *p* values had a probability level above 0.05, the threshold for statistical significance.

**Table 3:** Comparison of mean RMS and average values between four intervals for edentulous and dentate models separately

Factor	Group		Baseline vs. One-day	Baseline vs. fourteen days	Baseline vs. twenty-eight days
RMS	Edentulous	Intervals (*p* value)	0.251	0.002	0.002
	Dentate	Intervals vs. groups		0.921	0.536
Average	Edentulous	Intervals (*p* value)	0.954	0.209	0.321
	Dentate	Intervals vs. groups		0.065	0.056

## Discussion

Environmental factors cause a series of changes in the chemical composition and structure of polymer materials as they age. These alterations can change the material’s properties [11]. In addition, aging causes the leaching of resin components and accelerates the water absorption and solubility process, expansion, and degradation of the resin matrix, ultimately affecting the properties of printed resins [12].

This in vitro study aimed to assess the dimensional stability of 3D-printed edentulous and fully dentate hollowed maxillary models printed by the DLP lab technique over 1 day, 14 days, and 28 days using surface matching software. The printed models were kept in dry storage conditions at room temperature.

The outcome of the study showed that the RMS value of the models of edentulous patients moved up significantly from 0.257 to 0.368 within fourteen days and increased up to 0.384 in twenty-eight days. However, the RMS values were quite established at approximately 0.35 for the dentate models. Furthermore, the mean RMS values of the models of edentulism at the four intervals were evenly divided along with the RMS values of the models of dentate patients. That is why the effects of group (edentulous/dentate) became insignificant.

In addition, the curves of the mean values of the edentulous and dentate models move in different directions. Therefore, there was an interaction effect between the groups and the timeline. Hence, repeated-measures ANOVA was used separately for the observations related to edentulous data and dentate data. Those two analyses showed that mean RMS values increased insignificantly to the one-day interval but further increased significantly to fourteen days and twenty-eight days. However, the mean model values of dentate patients varied insignificantly among the four scans.

The observations of averages showed no significant differences between the timelines of edentulous, dentate or total models, as shown by repeated-measures ANOVA for combined and separated data, as discussed in the results section.

The mean values for edentulous patients increased from 0.014 to 0.029 over a period of twenty-eight days compared to the initial value. During this time period, however, the mean average values for the dentate models decreased from 0.033 to 0.014.

Teng Ma et al. [13] examined the influence of internal structures and different printing processes. They examined full-arch preparatory digital models. These models were solid, hollow, and grid interior designs. They found out that different internal structures significantly affected 3D printed resin model accuracy, whereas hollow models showed less accuracy regardless of the printing system. Shin et al. [5] and Yang and Huang [14] examined the impact of the internal structure on the precision of a 3D printed dental model. According to their statement, a model with an internal honeycomb structure and an object with the right amount of porosity can achieve excellent stability and accurate printing results. This also leads to greater mechanical properties, such as stability and strength, which aligns with the findings of Melchels et al. [15]. The utilization of a hexagon-filled model offers the benefit of minimizing resin usage. However, the detailed modelling procedure involved in designing its internal structure does not yield superior accuracy in the assessment of its precision. Consequently, it appears that there are limited advantages in employing this particular model. Although authors did come up to other studies of the impact of presence of teeth on the dimensional stability of aged model, it can be inferred that bulk and anatomy of teeth may delay the aging process. This point could be a field of further researches.

Despite the fact that statistical analysis revealed differences in 3 to 4 weeks of aging, the clinical significance of these findings remains unclear. Especially, when the reconstruction of the prostheses can be finalized on the model within 3 or 4 weeks and the models were been appropriately stored. Joda et al. revealed similar findings under constant conditions at 20 °C and 50% humidity without direct light exposure [1]. It needs to be emphasized that the changes in dimension of the 3D-printed models were slight and comparable to studies evaluating the accuracy of dental stone casts produced by traditional impression making [5, 13]. . Another vitro study compared conventional and 3D-printed dental cast accuracy and reproducibility. Polyetherketoneketone was used to build a master model. Ten Type IV dental stone with polyvinyl siloxane specimens were made. Three types of 3D printers (PolyJet, UV LED Digital light processing, and UV Digital light processing) produced 10 specimens each. The conventional casts changed volume less than the 3D-printed ones. Significant differences were observed among various 3D printer types. UV-polymerizing polymer with digital light processing had the smallest volumetric change. They found out that there were significant differences observed across different groups of models, and their impact did not seem to be clinically relevant [16]. Also, Hwang et al. [17] and Yoon et al. [18] studied the accuracy and adaptation of tissue surface of milled maxillary denture bases. The used digital light processing technique. They concluded that the clinical applicability of an error range of less than 100 μm has been confirmed. Hence, when considering the clinical significance of dimensional changes it is crucial to take into account the specific type of prostheses and the required duration for their fabrication.

One of the limitations of this study is that the models were investigated for a limited period up to 28 days. In an ideal situation, dental master castings for the production of retrievable prosthetic fabrications, such as screw-retained implant FPDs or removable prostheses, can be reused in longer time in the event of a future emergency. Other literature showed that the dimensional integrity of 3D-printed models subjected to testing decreased continuously over time. Lin et al. [19]assessed the influence of storage utilizing two printing techniques (SLA and DLP systems). They examined the solid designed models at two and six weeks. They concluded that the accuracy of the models was affected by storage for up to 6 weeks after printing. It could be inferred from this study that dimensions of hollowed models maybe subjected to further changes with time. However, since our study was for 28 days, we cannot exactly predict with certainty whether these changes will persist or stabilize over time. Therefore, until further investigations revealed the opposite, findings of this study suggested that 3D-printed dental master models should be regarded as single-use items, at least for the production of definitive prosthetic reconstructions during the studied time period.

Another limitation of this study is that the experiment focused only on the maxillary typodont model without studying also the mandibular one. The findings and recommendations could be totally different for the mandible as there are several dissimilarities between the two arches. Therefore, this topic has potential for more exploration, and is open for future research.

In general, caution must be exercised when translating in vitro findings into clinical performance. Since this study was conducted on a digital scan of the upper jaw, the consideration of oral digital impressions may vary with the mandible. The mobility of the mandible differs during dynamic movements. It might be difficult to transfer laboratory results from full-arch scans to in vivo patient situations, particularly in the mandible [1, 19]. 

## Conclusion

The study revealed changes in the dimensions of 3D-printed edentulous models as a result of the aging process over a span of 3 and 4 weeks. Based on the above results, it can be inferred that the use of 3D-printed dental master models for the production of definitive prosthetic reconstructions should be approached with caution if the time elapsed since 3D printing exceeds a period of 3 to 4 weeks. Nevertheless, the observed alterations associated with aging in this investigation were minimal and similar to the variability reported in traditional plaster cast models commonly utilized in ordinary clinical practices at present.

## Data Availability

The datasets used and/or analysed during the current study are available from the corresponding author on reasonable request.

## Footnotes

1 Repeated measures analysis of variance (ANOVA) is used to examine changes in mean scores over three or more time points.

## References

[1] Joda T, Ferrari M, Gallucci GO, Wittneben JG, Brägger U. Digital technology in fixed implant prosthodontics. Periodontol. 2000 (2017);(73):178 – 92. 10.1111/prd.12164 28000274

[2] Alghazzawi (2016). Advancements in CAD/CAM technology: options for practical implementation. J Prosthodont Res.

[3] Almufleh B, Emami E, Alageel O, de Melo F, Seng F, Caron E, Nader SA, Al-Hashedi A, Albuquerque R, Feine J (2018). Patient satisfaction with laser-sintered removable partial dentures: a crossover pilot clinical trial. J Prosthet Dent.

[4] Quan H, Zhang T, Xu H, Luo S, Nie J, Zhu X (2020). Photo-curing 3D printing technique and its challenges. Bioact Mater.

[5] Shin SH, Lim JH, Kang YJ, Kim JH, Shim JS, Kim JE. Evaluation of the 3D printing accuracy of a dental model according to its internal structure and cross-arch plate design: an in vitro study. Mater (2020);(13):5433. 10.3390/ma13235433 PMC7729473 33260676

[6] Hazeveld A, Huddleston Slater JJ, Ren Y (2014). Accuracy and reproducibility of dental replica models reconstructed by different rapid prototyping techniques. Am J Orthod Dentofac Orthop.

[7] Jeong YG, Lee WS, Lee KB (2018). Accuracy evaluation of dental models manufactured by CAD/CAM milling method and 3D printing method. J Adv Prosthodont.

[8] Lin LH, Granatelli J, Alifui-Segbaya F, Drake L, Smith D, Ahmed KE. A proposed in Vitro Methodology for assessing the Accuracy of three-dimensionally printed Dental models and the impact of Storage on Dimensional Stability. Appl Sci (2021); (13):5994.

[9] Joda T, Matthisson L, Zitzmann NU. Impact of aging on the accuracy of 3D-printed dental models: an in vitro investigation. J Clin Med (2020); (9): 1436. 10.3390/jcm9051436 PMC7291208 32408618

[10] Yousef H, Harris BT, Elathamna EN, Morton D, Lin WS. Effect of additive manufacturing process and storage condition on the dimensional accuracy and stability of 3D-printed dental casts. J Prosthet Dent (2022); (128):1041–6. 10.1016/j.prosdent.2021.02.028 33785200

[11] Tian Y, Chen C, Xu X, Wang J, Hou X, Li K, Lu X, Shi H, Lee ES, Jiang HB. A review of 3D printing in dentistry: technologies, affecting factors, and applications. Scanning. (2021). 10.1155/2021/9950131 PMC8313360 34367410

[12] Gad MM, Alshehri SZ, Alhamid SA, Albarrak A, Khan SQ, Alshahrani FA, Alqarawi FK. Water sorption, solubility, and translucency of 3D-printed denture base resins. J Dent (2022); (10):42. 10.3390/dj10030042 PMC8947006 35323244

[13] Ma T, Peng T, Lin Y, Zhang M, Ren G (2023). Effect of internal structures on the accuracy of 3D printed full-arch dentition preparation models in different printing systems. J Adv Prosthodont.

[14] Yang MY, Huang JS. Numerical analysis of the stiffness and strength of regular hexagonal honeycombs with plateau borders. Compos Struct (2004);(1):107–14.

[15] Melchels FP, Bertoldi K, Gabbrielli R, Velders AH, Feijen J, Grijpma DW. Mathematically defined tissue engineering scaffold architectures prepared by stereolithography. Biomaterials (2010);(27):6909–16. 10.1016/j.biomaterials.2010.05.068 20579724

[16] Valente Vda S, Zanetti AL, Feltrin PP, Inoue RT, de Moura CD, Padua LE. Dimensional accuracy of stone casts obtained with multiple pours into the same mold. ISRN Dentistry. (2012), 730674. 10.5402/2012/730674 PMC3540701 23320186

[17] Hwang H-J, Lee SJ, Park E-J, Yoon H-I (2018). Assessment of the trueness and tissue surface adaptation of CAD-CAM maxillary denture bases manufactured using digital light processing. J Prosthet Dent.

[18] Yoon H-I, Hwang H-J, Ohkubo C, Han J-S, Park E-J (2018). Evaluation of the trueness and tissue surface adaptation of CAD-CAM mandibular denture bases manufactured using digital light processing. J Prosthet Dent.

[19] Lin LH, Granatelli J, Alifui-Segbaya F, Drake L, Smith D, Ahmed KE (2021). A proposed in Vitro Methodology for assessing the Accuracy of three-dimensionally printed Dental models and the impact of Storage on Dimensional Stability. Appl Sci.

